# A Review of the Current Clinical Aspects of Sjögren’s Disease: Geographical Difference, Classification/Diagnostic Criteria, Recent Advancements in Diagnostic Methods, and Molecular Targeted Therapy

**DOI:** 10.3390/jcm14155577

**Published:** 2025-08-07

**Authors:** Yoshiro Horai, Shota Kurushima, Toshimasa Shimizu, Hideki Nakamura, Atsushi Kawakami

**Affiliations:** 1 Department of Rheumatology, Sasebo City General Hospital, Sasebo 857-8511, Japan; 2 Department of Immunology and Rheumatology, Division of Advanced Preventive Medical Sciences, Nagasaki University Graduate School of Biomedical Sciences, Nagasaki 852-8523, Japan; 3 Clinical Research Center, Nagasaki University Hospital, Nagasaki 852-8501, Japan; 4 Division of Hematology and Rheumatology, Department of Medicine, Nihon University School of Medicine, Tokyo 173-8610, Japan

**Keywords:** artificial intelligence, biological drugs, classification criteria, diagnostic criteria, guidelines, Janus kinase inhibitors, recommendations, Sjögren’s Disease

## Abstract

Sjögren’s Disease (SjD) is an autoimmune disorder characterized by sicca symptoms arising from impaired salivary and lacrimal gland function and accompanying extraglandular involvement. SjD is recognized as an illness of female dominance for which the 2002 American–European Consensus Group Classification Criteria and the American College of Rheumatology/European Alliance of Associations for Rheumatology 2016 classification criteria are utilized for inclusion in clinical trials, and treatment recommendations from countries belonging to the American College of Rheumatology or the European Alliance of Associations for Rheumatology are globally recognized. It is presumed that there are geographical differences among female sufferers, and unique diagnostic criteria and recommendations are used in clinical practice in Japan. In addition to the items included in the classification criteria, several methods to measure saliva secretion, serum biomarkers, and artificial intelligence tools have recently been reported to be useful for the assessment of SjD. While symptomatic therapies including tear drops, artificial saliva, and muscarinic agonists are still the mainstay for treating SjD, several kinds of molecular targeted drugs, such as biological drugs and Janus kinase inhibitors, that are expected to improve the prognosis of SjD have been tested in recent clinical trials.

## 1. Introduction

Sjögren’s Disease (SjD) which was previously referred to as Sjögren’s syndrome (SS) but has been renamed to better communicate its seriousness [1], is an autoimmune disorder characterized by sicca symptoms arising from impaired salivary and lacrimal gland function and accompanying extraglandular involvement. It is presumed to have a multifactorial pathophysiology involving viral infections, innate immunity, and epigenetic mechanisms [1,2,3]. SjD is often considered a mild disease, with dry eyes and mouth hinting toward a diagnosis; however, these sicca symptoms might not always be an initial manifestation of the disease and may be the result of its progression instead. Relying only on sicca symptoms might lead to a delayed diagnosis of SjD [4,5]. In addition, SjD is not always a mild disease, and it sometimes affects life expectancy. In a study involving a Spanish population, patients with SjD and a European Alliance of Associations for Rheumatology (EULAR) SS Disease Activity Index (ESSDAI) [6] of 14 or more at diagnosis were at a risk of worse mortality [7]. According to previous studies, patients with SjD are at risk of developing complications including other rheumatic diseases, such as rheumatoid arthritis (RA), systemic lupus erythematosus (SLE) [8], and dementia [9]. It is widely known that patients with SjD are at a high risk of developing complicating lymphoma. In addition to lymphoma, risks of other types of malignancies, such ovarian and small bowel cancer, were reported to be elevated in patients with SjD [10]. There is currently no established disease-modifying therapy for SjD, and patients with SjD are at risk of complications like lymphoma, for which chemotherapy is necessary. Additionally, complication rates of hematological abnormalities, lung manifestations, and renal tubular acidosis were reported to be positively correlated with disease duration in SjD, highlighting the importance of early diagnosis in SjD [11]. Nevertheless, diagnosis of SjD tends to be delayed due to several factors such as non-specific initial symptoms, insufficient awareness of the disease, and insufficient health insurance infrastructure, which varies among countries and might hinder early referral from general practitioners to specialists [5]. With the above-mentioned risks of complications in patients with SjD, including lymphoma, early diagnostic strategies that result in the initiation of treatment for patients with SjD within an appropriate period require improvement.

The 2002 American–European Consensus Group (AECG) SS Classification Criteria [12] and the American College of Rheumatology (ACR) and EULAR 2016 classification criteria [13,14] are globally accepted criteria for the diagnosis of SjD and have been utilized as inclusion criteria in studies of SjD, even in Japan. Clinical guidelines for the diagnosis and treatment of rheumatic diseases have been mainly published in countries [12,13,14,15,16,17,18,19,20] that belong to the ACR or EULAR, and SjD is no exception. However, diagnostic criteria and practice guidelines unique to Japan have been developed [21,22] and are still utilized in current clinical practice in Japan. In addition, as noted below, new methods for assessing secretion function and diagnostic imaging for SjD have recently been reported by Japanese researchers.

In this review, we detail the current aspects of the diagnosis and treatment of SjD, including recent findings reported from Japan and a comparison of clinical practice in Japan and other countries. A transition from the term ‘SS’ to ‘SjD’ is taking place, so we have used ‘SS’ where necessary, considering the publication date of the relevant studies. SjD is categorized as primary or secondary based on the presence or absence of other rheumatic diseases [12,23]. In the following sections, we refer to primary SjD as ‘SjD’ unless otherwise stated.

## 2. Epidemiology of SjD: Differences Among Countries/Continents

SjD is widely recognized as a female-dominant disease, similar to other rheumatic diseases such as SLE. According to a systemic literature review conducted by Thurtle et al. from a global perspective [24], the proportion of females in the total number of SjD cases ranged from 51 to 90% in the studies considered in their analysis. Three studies from European countries or the United States also found female dominance. In Australia, which is located in Oceania, the dominance of females among SjD patients was found to be 90.1% in a study of a south Australian cohort [25]. Considering studies conducted in Africa, the dominance of females among patients with SjD was 77.27% (in a sub-analysis limited to primary SjD) in a study (in which both primary SjD and secondary SjD were present) in Egypt [26]. According to a nationwide survey conducted in Japan, the dominance of females among patients with SjD was 94.2%, which is very different from the above-mentioned result of 51 to 90% from Thurtle et al. [24]. It should be noted that 58.5% of patients were diagnosed as having primary SjD, and 39.2% of patients were diagnosed with secondary SjD (the details were not clear for the other 2.3% of patients) [27]. While limitations such as patient numbers and study periods, which differed between studies, should be noted, it is possible that the dominance of females among SjD patients may vary depending on ethnicity. According to a recent analysis of hospitalizations of patients with SjD in a Polish population by Domańska-Poboża et al., female SjD patients were older than male SjD patients and had a longer admission period, whereas male SjD patients had slightly higher mortality compared to females. Although further investigation into immunological and hormonal differences seems necessary for clarification purposes, SjD in males might represent a different phenotype than SjD in females [28].

## 3. Classification/Diagnostic Criteria for SjD

The criteria for SjD published in distinguished journals are utilized for classification and for inclusion in SjD clinical trials, whereas the above-mentioned Japanese criteria are categorized as ‘for diagnosing’ [21]. The above-mentioned 2002 AECG criteria [12] have been widely applied as inclusion criteria in clinical analyses of SjD. In 2012, Sjögren’s International Collaborative Clinical Alliance (SICCA) advocated classification criteria including three objective items, anti-Ro/SS-A antibodies and anti-La/SS-B antibodies, labial salivary gland biopsy, and ocular staining score, and considered developing these criteria for use as enrollment criteria in clinical trials for the development of biological therapy [29]. Thereafter, the ACR and EULAR announced revised classification criteria for SS as a collaborative project in 2016 [13,14]. While the 2002 AECG criteria [12] are the most commonly used [13,14] and are still referenced, depending on study protocols, the 2016 ACR/EULAR classification criteria are frequently adopted in current clinical studies of SjD. The 2016 ACR/EULAR classification criteria are utilized even for clinical studies conducted in Japan; however, the criteria for SS proposed by the Japan Research Committee of the Ministry of Health, Labour, and Welfare (JMHLW) in 1999 [21] have also been adopted for the diagnosis of SjD, which the Japanese government designates as an intractable disease. The 1999 JMHLW diagnostic criteria consist of four major items: histopathology obtained from salivary or lacrimal gland biopsy, oral examination by sialography or saliva secretion tests in combination with salivary gland scintigraphy, Schirmer’s test in combination with ocular staining test with rose Bengal or fluorescein, and autoantibodies, such as anti-Ro/SS-A antibodies and anti-La/SS-B antibodies. Different from the 2002 AECG criteria and the 2016 ACR/EULAR classification criteria, subjective symptoms such as dry eye and dry mouth were not considered prerequisite or classification items in the 1999 JMHLW diagnostic criteria, with the aim of developing criteria that enable diagnosis via objective examination without a requirement for subjective symptoms [21]. Consistencies among the 2002 AECG criteria, the 2012 SICCA criteria, and the 1999 JMHLW diagnostic criteria in the Japanese population have previously been examined, and the results indicate better sensitivity compared to the 2002 AECG criteria and the 2012 SICCA criteria, comparable specificity to the 2002 AECG criteria, and better specificity compared to the 2012 SICCA criteria of the 1999 JMHLW diagnostic criteria [30]. Validation of the 2016 ACR/EULAR classification criteria in a Japanese population was also performed: the 2016 ACR/EULAR classification criteria showed higher sensitivity but lower specificity compared to the 2002 AECG criteria, the 2012 SICCA criteria, and the 1999 JMHLW diagnostic criteria [31].

Sicca symptoms are the most well-known symptoms of SjD, but tear and saliva secretion tests are also included in the 2002 AECG criteria, 2016 ACR/EULAR classification criteria, and 1999 JMHLW diagnostic criteria. To assess tear secretion, Schirmer’s test is used in the 2002 AECG criteria, 2016 ACR/EULAR classification criteria, and 1999 JMHLW diagnostic criteria.

Although saliva secretion tests are included as classification items in the 2002 AECG criteria, the 2016 ACR/EULAR criteria, and the 1999 JMHLW diagnostic criteria, the methods used to quantify saliva vary among the criteria. While unstimulated whole salivary flow is included in the 2002 AECG criteria and the 2016 ACR/EULAR criteria, decreased results of stimulated salivary flow obtained via the chewing gum test or Saxon test in combination with salivary dysfunction on scintigraphy are included in the 1999 JMHLW diagnostic criteria. However, both unstimulated and stimulated saliva secretion tests take time, and precautions against infection related to contact with saliva are required in the COVID-19 era. Sakamoto et al. investigated the utility of an oral moisture meter, a method used to measure water content in oral mucosa in a few seconds, which was developed by a Japanese medical equipment manufacturer, in patients with SjD who were admitted to the department of oral surgery of a Japanese university hospital with which the investigators were affiliated. They found equivalent sensitivity, specificity, and accuracy when using the oral moisture meter and assessing unstimulated whole salivary flow, the chewing gum test, and the Saxon test, the results of which suggested some degree of liability when adopted as a criterion for the diagnosis of SjD [32]. Noguchi et al. applied machine learning, a subset of artificial intelligence (AI), to analyze tongue photographs in an attempt to detect SjD via a noncontact method [33]. These methods are preliminary and needed to be studied in greater numbers of patients.

As noted above, salivary gland scintigraphy is included in the 2002 AECG criteria [12]. However, this method, which involves exposure to radiation and a complicated procedure, is not included in the 2016 ACR/EULAR classification criteria [13,14,34]. On the other hand, salivary gland scintigraphy is useful for the diagnosis of SD in patients in whom labial salivary gland biopsy is impossible and is still utilized in the 1999 JMHLW diagnostic criteria in clinical practice in Japan. Notably, in the 1999 JMHLW criteria, the requirement for positive findings of SjD is described as ‘decreased salivary function’, i.e., the criteria for assessing findings of salivary gland scintigraphy might vary among facilities, and uptake patterns in salivary glands characteristic of SjD are not clarified. Recently, Inokuma et al. analyzed findings of uptake in salivary glands in sialoscintigraphy in patients who visited a hospital in Japan and were suspected to have SjD without thyroid dysfunction; they found less uptake in the submandibular glands compared to the parotid glands [35].

Labial salivary gland biopsy, the significance of which is considered very important in the diagnosis of SjD, is included in all of the above criteria. However, the sensitivity of labial salivary gland biopsy might be altered by disease stage, potentially leading to false negative results [36]. Tryposkiadis et al. recently reported that the number of minor salivary glands was pathologically associated with positive findings for a diagnosis of SjD; five or more were ideal for obtaining a focus score ≥ 1 [37].

Anti-Ro/SS-A antibodies and anti-La/SS-B antibodies are well-known autoantibodies found in patients with SjD. Anti-Ro/SS-A antibodies, autoantibodies detected in two-thirds of patients with SjD [38], are included in all of the four classification/diagnostic criteria for SjD [12,13,14,21,29], whereas anti-La/SS-B antibodies, the isolated positive detection of which was reported as not useful for the diagnosis of SjD [39] and even other autoimmune diseases [38], are not included in the 2016 ACR/EULAR classification criteria [13,14]. Rheumatoid factor (RF) and antinuclear antibody (ANA) detection is included in the classification criteria for RA [40] and SLE [41], respectively. In patients with SjD, positive rates of RF and ANA were reported to range from 38 to 61% and from 50 to 89%, respectively [42]. Therefore, precise diagnostic procedures, including differential diagnosis between RA/SLE and SjD or confirmation of the coexistence of RA/SLE and SjD, should be performed. The positive detection of rheumatoid factors in combination with antinuclear antibodies is a classification item in the 2012 SICCA criteria [29]. These four autoantibodies might be useful for both diagnosing and stratifying SjD. Bodakçi conducted an analysis of 402 patients with SjD who were divided into three groups based on the positive detection of anti-Ro/SS-A antibodies, anti-La/SS-B antibodies, RF, and ANA. In this analysis, the patient group positive for all four autoantibodies was significantly younger at diagnosis compared to the patient group positive for anti-Ro/SS-A antibodies and ANA. Moreover, there were statistically significantly higher complication rates of physical manifestations including lymphadenopathy; Raynaud’s phenomenon; vasculitis; neurological involvement; arthritis; organ involvement, including interstitial lung disease and autoimmune thyroiditis; and hematological abnormalities, including cytopenia and hypergammaglobulinemia, in the patient group positive for all four autoantibodies compared to the patient group positive for anti-Ro/SS-A antibodies and ANA and the patient group positive for none of the four antibodies [42]. These results suggest that clinical phenotypes of SjD could be classified depending on autoantibody profiles, which suggest the priority of autoantibody profiling for phenotype stratification. Differences between the 2002 AECG criteria, the 2016 ACR/EULAR classification criteria, and the 1999 JMHLW diagnostic criteria, are summarized in Table 1.

## 4. Assessment of Disease Activity and Prognosis for SjD: Composite Measure (ESSDAI), Prospective Biomarkers, Diagnostic Images, and Artificial Intelligence

The ESSDAI [6] is a widely recognized tool for assessing disease activity in SjD and is adopted as a criterion of medical expense subsidies for patients with SjD in Japan. In the ESSDAI, a calculation weight is assigned for each component described in Table 2, for example, ′5′ for renal and ′2′ for glandular [6]. The utility of the ESSDAI is well established, and high ESSDAI scores of 14 or more at diagnosis have been reported to be associated with worse mortality, as mentioned above [7]. However, calculating this composite measure is not always easy in daily clinical practice. The identification of a single biomarker reflecting systemic disease activity in SjD might contribute to efficient clinical practice regarding SjD. Zong et al. reported that hypergammaglobulinemia was identified as a risk factor for worse mortality in patients with SjD in a Chinese population [43]. Akiya et al. analyzed serum zinc concentrations in patients with SjD and patients with RA who visited Nihon University hospital (Tokyo, Japan), the authors’ affiliated hospital, and found a higher rate of zinc deficiency in patients with SjD compared to patients with RA. In addition, patients with an ESSDAI score of five or more showed a statistically significantly lower level of serum zinc concentration than patients with an ESSDAI score of four or less [44], which might be partly attributable to organ involvement in SjD.

Among extraglandular manifestations, kidney involvement, including renal tubular acidosis, might result in end-stage renal disease [45]. In addition to renal tubular acidosis, nephrolithiasis and nephrocalcinosis have been identified as factors of renal dysfunction in SjD [46]. Huang et al. retrospectively analyzed the characteristics of 1293 patients with SjD, including the results of hypertension, blood, and urine analyses and Chisholm and Mason scoring. Based on the results, they developed a nomogram which could be useful for the early detection and treatment of renal involvement [47].

Despite not being included in the classification/diagnostic criteria mentioned in this article, advantages of ultrasound (US), magnetic resonance imaging, and computed tomography in assessing damage to major salivary glands in SjD have been reported [34]. US is a non-invasive, radiation-free method used to observe internal organs, the usefulness of which is reportedly applicable to diagnostic imaging of major salivary glands in SjD. Recently, Japanese rheumatologists reported patients with SjD who demonstrated improvements in US findings after immunosuppressive therapy, including glucocorticoid therapy (GC) [48,49], suggesting the usefulness of US in assessing treatment response. US could be beneficial for assessing salivary glands and lacrimal glands. Salbas et al. recently reported the utility of microvascular imaging for detecting vascularity on lacrimal glands [50].

Current progress in AI has the potential to dramatically improve the accuracy of diagnostic imaging, which might be applied to SjD. Kise et al. reported preliminary results showing that deep learning, a subset of machine learning which was applied to analyze tongue photographs of SjD, as mentioned above [33], showed diagnostic accuracy on CT findings of SjD parotid glands comparable to that of experienced radiologists [51].

## 5. Treatment of SjD: Current Situation

The treatment guidelines developed by the Sjögren’s Syndrome Foundation (SSF), targeting SjD in the United States [15,16,17,52], and management recommendations developed by the EULAR [18] are currently available. Other than these recommendations/guidelines, the British Society for Rheumatology recently announced guidelines on the management of adult- and juvenile-onset SjD [19]. In Japan, a 2017 clinical practice guideline was developed as part of a JMHLW research program for intractable diseases [22]. A comparison of the clinical aspects, including classification/diagnostic criteria and treatment recommendations/guidelines for SjD among countries belonging the ACR or EULAR and Japan, is summarized in Table 3.

Symptomatic therapies including tear drops, artificial saliva, and muscarinic agonists are still the mainstay for treating SjD [53]. These therapies remain unchanged in current treatment recommendations/guidelines, even with the development of molecular targeted therapy such as biological drugs and Janus kinase inhibitors (JAKis), which have revolutionized therapies for other rheumatic diseases such as RA and SLE. Oral analgesics might be an option for relieving SjD-related neuropathic ocular pain, which can persist even after local treatment. Tagawa et al. recently reported a Japanese female patient with complicated ocular pain associated with SjD that persisted even after improvements in objective findings following treatment with various kinds of eye drops and punctal plugs; among these, mirogabalin was effective at alleviating pain [54]. Although mirogabalin can relieve pain, it has no disease-modifying immunosuppressive effects. Applications of immunomodulators/immunosuppressants including molecular targeted drugs have been proposed as potential options in guidelines/recommendations for SjD published in recent years and subsequent clinical studies.

Hydroxychloroquine (HCQ) is an analog of chloroquine, the effects of which were mentioned in recommendations for rheumatic diseases such as RA and SLE [55,56,57]. Although the therapeutic effects of HCQ on tear and saliva secretion were not elucidated in previous clinical studies [18], HCQ was introduced in selected situations to treat fatigue in SjD as part of the SSF treatment guideline [17]. A trial administration of HCQ for patients with SjD with fatigue and systemic symptoms is also suggested in the 2024 British Society for Rheumatology guidelines for the management of SjD [19], although no statistical difference in fatigue was found following treatment with HCQ in the management recommendations developed by the EULAR [18]. As noted in the Introduction, patients with SjD are reported to have a high risk of developing dementia [9]. In a study by Lee et al., the administration of HCQ was associated with a lower rate of dementia in patients with SjD [9]. In the SSF treatment guideline, GC was listed as a treatment option for musculoskeletal pain in SjD, whereas GC-sparing agents should be considered for long-term administration [17]. Recently, the administration of HCQ in combination with leflunomide was shown to be associated with reduced ESSDAI [58], which was attributable to effects targeted at type I interferon-associated proteins that resulted in a decrease in B cell activity [59].

## 6. Treatment of SjD: Potential Molecular Targeted Therapy

Considering the effectiveness of molecular targeted drugs, which has been shown to be much higher than that of conventional immunosuppressive drugs in other rheumatic diseases, there are high expectations for their use in SjD. Although strong recommendations have not been made for the use of molecular targeted drugs in published recommendations/guidelines, several types of biological drugs indicated for other rheumatic diseases have been tested for the treatment of SjD. Improvements in disease activity at 48 weeks of treatment with abatacept, a biological disease-modifying antirheumatic drug classified as cytotoxic T-lymphocyte-associated protein-4 immunoglobulin, were observed in the ASAP-III trial [60]. In a phase II trial, combination therapy consisting of belimumab (BLM) and rituximab (RTX), which act as a B-cell-activating factor inhibitor and an antibody against CD20, respectively, was shown to reduce the ESSDAI compared to placebo and demonstrated safety comparable to that of monotherapy [61]. Watanabe et al. reported a Japanese male patient with TAFRO syndrome and SjD, which was refractory to high-dose GC and tocilizumab but successfully treated with BLM and RTX. In their report, the authors discussed the effectiveness of combination therapy consisting of BLM and RTX in treating TAFRO syndrome associated with SjD from the perspective that TAFRO syndrome might be a severe form of SjD [62]. Telitacicept, which blocks B-cell-activating factor and a proliferation-inducing ligand, has been reported to reduce the ESSDAI compared to placebo in a phase II trial [63]. Recently, the effectiveness of telitacicept in SjD in actual clinical practice was reported in a case series in which the exclusion criteria were the use of other biological drugs or incomplete follow-up; six out of seven patients who were treated with telitacicept in China achieved improvements in the ESSDAI [64].

In a study of labial salivary glands involving patients with SjD and healthy individuals who were admitted to Tokushima University Hospital (located on Shikoku Island, Japan), JAK1 and JAK2 were found to be strongly expressed in the ductal and acinar cells of labial salivary glands from patients with SjD, and the effects of baricitinib (a type of JAKis which has specificity for JAK1 and JAK2) on suppressing interferon-gamma-induced CXCL10 expression through the inhibition of the JAK/STAT pathway were shown [65]. While the clinical effects of JAKis on SjD are still being validated, a phase Ib/IIa randomized controlled trial of tofacitinib, another type of JAKi, in SjD has been planned based on experimental results revealing the inhibitory effects of tofacitinib on Type I interferon response in salivary gland epithelial cells [66]. Recently, the therapeutic effects of tofacitinib in improving the ESSDAI were shown in a study conducted in China, which comprised a retrospective cohort and prospective cohort. In that study, the effects of tofacitinib were influenced by different types of concomitant immunosuppressants/immunomodulators [67]. Further studies are needed to clarify the effects and drug interactions of tofacitinib in SjD. Potential molecular targeted therapy for SjD is summarized in Table 4.

Other than the above-mentioned drugs, recently introduced drugs such as nipocalimab, efgartigimod, dazodalibep, and deucravacitinib has been tested in clinical trials for SjD. Among these candidates, dazodalibep was shown to decrease the ESSDAI or EULAR Sjögren’s Syndrome Patient Reported Index in a phase 2 trial [68]. As for deucravacitinib, a phase-2 study of its effect on SjD is ongoing [69]. It is presumed that the molecular targeted drugs listed above could revolutionize treatment for SjD, but their high cost necessitates a cost-effectiveness evaluation. The increased prevalence of biosimilar drugs for the treatment of SjD could prove helpful. Evidence for the treatment of juvenile SjD is limited [19], requiring further development.

## 7. Conclusions

The common theory that SjD mainly affects females is almost applicable globally; however, there may be differences in rates depending on ethnicity, and SjD in males might be a different phenotype than SjD in females. Sicca symptoms are the primary symptoms of SjD, but they may not be the initial symptoms. Moreover, SjD is heterogenous in terms of severity and complications. Therefore, physicians should assess patients cautiously, looking not only for sicca symptoms but also possible organ complication. The application of diagnostic imaging in combination with AI might be a solution for the early detection of SjD lesions. Although symptomatic therapies are still the mainstay for the treatment of SjD, recent advancements in molecular targeted therapy might result in a paradigm shift in the treatment of SjD. Clinical development of molecular targeted therapy based on further studies is expected to contribute to improving prognosis in SjD.

## Figures and Tables

**Table 1:** Differences 2002 AECG criteria, 2016 ACR/EULAR classification criteria, and 1999 JMHLW diagnostic criteria for SjD.

	The 2002 AECG Criteria	The 2016 ACR/EULAR Classification Criteria	The 1999 JMHLW Diagnostic Criteria
Subjective sicca symptoms	Adopted as classification items	Described as inclusion criteria	Neither adopted as a prerequisite nor classification items
Tear secretion test	Schirmer’s test		
Ocular staining	van Bijsterveld score using rose bengal	Ocular staining score (using lissamine green and fluorescein); van Bijsterveld score using rose bengal	van Bijsterveld score using rose bengal Fluorescein staining test
Saliva secretion test	Unstimulated whole saliva flow rate		Chewing gum test or Saxon test
Diagnostic imaging	Sialography Salivary gland scintigraphy	None adopted	Sialography Salivary gland scintigraphy *
Biopsy	Minor salivary gland		Minor salivary gland and/or lacrimal gland
Serum autoantibody	Ant-Ro/SS-A Ab and/or anti-La/SS-B Ab	Ant-Ro/SS-A Ab	Ant-Ro/SS-A Ab and/or anti-La/SS-B Ab

Ab, antibody; AECG, American–European Consensus Group; ACR, American College of Rheumatology; EULAR, European Alliance of Associations for Rheumatology; SjD, Sjögren’s Disease; JMHLW, Japanese Ministry of Health, Labor, and Welfare; * scored based on decreased saliva secretion test.

**Table 2:** Weights for each component included in ESSDAI.

Domain	Weight
Constitutional	3
Lymphadenopathy	4
Glandular	2
Articular	2
Cutaneous	3
Pulmonary	5
Renal	5
Muscular	6
Peripheral nervous system	5
Central nervous system	5
Hematological	2
Biological	1

**Table 3:** Comparison of clinical aspects including classification/diagnostic criteria and treatment recommendations/guidelines for SjD among countries belonging ACR or EULAR and Japan.

	Countries Belonging to ACR or EULAR	Japan
Classification/diagnostic criteria	The 2002 AECG criteria, the 2016 ACR/EULAR classification criteria	In addition to the two criteria on the left, the 1999 JMHLW criteria are adopted for the diagnosis of SS, designated as an intractable disease by the Japanese government
Epidemiology	Female dominance	Potentially greater female dominance compared to countries belonging ACR or EULAR (94.2% *)
Recommendations/guidelines	SSF treatment guidelines EULAR management recommendations BSR management guideline	The 2017 clinical practice guidelines of the research program for intractable diseases of the JMHLW

AECG, American–European Consensus Group; ACR, American College of Rheumatology; BSR, British Society for Rheumatology; EULAR, European Alliance of Associations for Rheumatology; SjD, Sjögren’s Disease; SSF, Sjögren’s Syndrome Foundation; JMHLW, Japanese Ministry of Health, Labor, and Welfare; and * in the nation-wide survey in Japan which produced these results, the study patient group consisted of primary SjD, secondary SjD, and 2.3% of unclassified patients.

**Table 4:** Potential molecular targeted therapy for SjD.

Biological Drugs	References
Abatacept	[60]
(cytotoxic T-lymphocyte-associated protein-4 immunoglobulin)	
Belimumab (B-cell-activating factor inhibitor) and	[61,62]
Rituximab (antibody against CD20)	
Telitacicept	[63]
(blocker of B-cell-activating factor and a proliferation-inducing ligand)	
Janus kinase inhibitor	
Tofacitinib	[66,67]

## Data Availability

Not applicable.
